# Establishment of virus-induced gene silencing (VIGS) system in *Luffa acutangula* using Phytoene desaturase (*PDS*) and tendril synthesis related gene (*TEN*)

**DOI:** 10.1186/s13007-023-01064-4

**Published:** 2023-08-31

**Authors:** Xiaoyu Qi, Qiaoping Mo, Jing Li, Zhibo Zi, Mengyun Xu, Suju Yue, Hongbo Zhao, Haisheng Zhu, Guoping Wang

**Affiliations:** 1 College of Horticulture, South China Agricultural University, Guangzhou, 510642 Guangdong China; 2 Key Laboratory of Biology and Germplasm Enhancement of Horticultural Crops in South China, Ministry of Agriculture, College of Horticulture, South China Agricultural University, Guangzhou, 510642 Guangdong China; 3 College of Plant Science, Tibet Agricultural and Animal Husbandry University, Tibet, 860000 Nyingchi China; 4 College of Life Sciences, Zhongkai University of Agriculture and Engineering, Guangzhou, 510225 Guangdong China; 5 Fujian Key Laboratory of Vegetable Genetics and Breeding/Crops Research Institute, Fujian Academy of Agricultural Sciences, Fujian 350013 Fuzhou, China

**Keywords:** *Luffa*, VIGS, Cucumber green mottle mosaic virus (CGMMV), *PDS*, *TEN*

## Abstract

**Background:**

Virus-induced gene silencing (VIGS) is a reverse genetics technology that can efficiently and rapidly identify plant gene functions. Although a variety of VIGS vectors have been successfully used in plants, only a few reports on VIGS technology in *Luffa* exist.

**Results:**

In the present study, a new cucumber green mottle mosaic virus (CGMMV)-based VIGS vector, pV190, was applied to establish the CGMMV-VIGS to investigate the feasibility of the silencing system for *Luffa*. Phytoene desaturase (*PDS*) gene was initially selected as a VIGS marker gene to construct a recombinant vector. Plants infected with *Agrobacterium* harboring pV190-*PDS* successfully induced effective silencing in *Luffa*, and an effective gene silencing phenotype with obvious photobleaching was observed. To further validate the efficiency, we selected *TEN* for gene-silencing, which encodes a CYC/TB1-like transcription factor and is involved in tendril development. *Luffa* plants inoculated with the pV190-*TEN* exhibited shorter tendril length and nodal positions where tendrils appear are higher compared to those of non-inoculated plants. RT-qPCR showed that the expression levels of *PDS* and *TEN* were significantly reduced in the CGMMV-VIGS plants. Moreover, we evaluated the CGMMV-VIGS efficiency in three cucurbits, including cucumber, ridge gourd, and bottle gourd.

**Conclusion:**

We successfully established a CGMMV-based VIGS system on ridge gourd and used marker genes to identify the feasibility of the silencing system in *Luffa* leaves and stems.

**Supplementary Information:**

The online version contains supplementary material available at 10.1186/s13007-023-01064-4.

## Background

Virus-induced gene silencing (VIGS) is a natural defense mechanism of plants against virus infection, and it is produced by silencing genes in plants after they have been invaded by viruses. It performs gene silencing at the post-transcriptional level. VIGS is used to construct a recombinant viral vector by inserting target gene fragments that can recognize a function into the appropriate viral genome and then use the recombinant viral vector to infect the plant to inhibit the endogenous gene of the plant, thereby inducing corresponding changes in the phenotypic or physiological traits of the plant. The mechanism of action of VIGS is that plants resist viral infection by recognizing and processing double-stranded RNA intermediates of viral replication [1, 2]. This results in sequence-specific RNA degradation, known as RNA silencing [3].

When host gene fragments are integrated into an engineered virus, the degradation of host-derived sequences, as part of the viral RNA, also targets the corresponding host mRNA for post-transcriptional silencing [4]. The potential of VIGS as a functional genomics tool was quickly recognized because it allowed targeted downregulation of specific host genes [5]. VIGS is a reverse genetics technology that can efficiently and rapidly identify plant gene functions. Compared with traditional gene function verification technologies such as gene knockout and transgenics, this technology has the advantages of simple operation, short cycle, low cost, and rapid phenotype acquisition, and it has wide application in plant genomics research.

VIGS has been used for functional identification of genes in many plant species. To date, at least 50 VIGS vectors based on RNA viruses, DNA viruses, and satellites have been developed for dicots and monocots [6]. Recently, VIGS-based technologies were developed to enable gene silencing and overexpression, such as virus-induced overexpression (VOX), genome editing virus-induced genome editing (VIGE), and host-induced gene silencing (HIGS) [7]. However, only a few VIGS studies on cucurbits have been reported, and the viral vectors are currently applied to cucurbit species, including apple latent spherical virus (ALSV) [8], tobacco ringspot virus (TRSV) [9], tobacco rattle virus (TRV) [10, 11], cucumber green mottle mosaic virus (CGMMV) [12] and cucumber fruit mottle mosaic virus (CFMMV) [13]. ALSV vectors were evaluated for the VIGS of *magnesium chelatase *(*SU*) and *phytoene desaturase *(*PDS*) genes among a broad range of plant species, including six cucurbit species [8]. TRSV-based gene silencing vector could knock down the expression of the endogenous gene *PDS* in melon, watermelon, cucumber and *N. benthamiana* [14]. CGMMV-based VIGS vector effectively silenced the *PDS* in watermelon, cucumber, and gourd, but very weakly photobleaching phenotypes in *N. benthamiana* [12]. Using the CFMMV-based VIGS vector pCF93, the silencing of eight of the 38 candidate genes related to male sterility in watermelon produced sterile male flowers with abnormal stamens and no pollen [13].

*Luffa* is an annual climbing herb of the Cucurbitaceae family. *Luffa* including two species: sponge gourd (*Luffa cylindrica*) and ridge gourd (*Luffa acutangula*). Ridge gourd is an important vegetable in south China and southeast Asia. With the completion of the whole genome sequencing of two *Luffa* species [15–17], research on the gene function of important traits has become imminent. The verification of gene function through genetic transformation in *Luffa* is difficult and time-consuming. Previous studies have reported that *Agrobacterium tumefaciens* mediated the genetic transformation of *L. cylindrica* but did not produce transformed plants [18, 19]. Therefore, the VIGS system in *Luffa* is an alternative method to the reverse genetic approach in the study of gene function. The system will help in the efficient and rapid identification of genes of interest [8].

CGMMV was reported to be associated with the leaf green mosaic disease of *Luffa acutangula* [20]. A system of CGMMV-based VIGS was recently developed, and its applicability to several cucurbits was verified [12]. Although the CGMMV-based VIGS system in cucurbits is established, its efficiency in ridge gourd remains unclear. In the present study, we used a CGMMV-based VIGS system in *L. acutangula* using *Phytoene desaturase *(*PDS*) and *tendril gene *(*TEN*) to investigate the feasibility of the silencing system in *Luffa* leaves and stems.

## Materials and methods

### Plant material

‘L422’, a cultivated material of *L. acutangula*, was used for this experiment. Seeds were soaked for 24 h in water, germinated at 30 ℃ under dark and humid conditions, and then sown in soil after radicle emergence. The plant trays were kept in a growth chamber with a 16 h light/8 h dark photoperiod at 28 ℃.

### Cloning of ***LaPDS*** and ***LaTEN*** partial sequence

We used *PDS* as a control marker gene and *TEN* as the target gene for the formation of tendrils to evaluate the efficiency of VIGS in *Luffa*. The cucumber *PDS* (*Csa4G011080*) and *TEN* (*Csa5G644520*) gene sequences were blasted against a local transcript data of ‘L422’ to obtain their orthologous sequences. The primers spanning about 300 bp cDNA regions were designed (Additional file: 1 Table. S1) The 50 µL PCR system contained Phanta Max Super-Findelity DNA polymerase 1 µL, template 2 µL, forward/reverse-primer (10 µM) 2 µL, dNTP Mix 1 µL, 2 Phanta^®^ Max buffer 25 µL. The PCR conditions were as follows: pre-denaturation at 95 ℃ for 30 min, followed by 95 ℃ for 30 s, annealing at 56 ℃ for 30 s, extension at 72 ℃ for 60 s for a total of 34 cycles, and a final extension at 72 ℃ for 5 min. The electrophoresis was performed on 1.0% agarose gel. The target fragment was purified according to the instructions of the HiPure Gel Pure DNA Mini Kit (Magen Biotech Co., Ltd, Guangzhou), and stored at − 20 ℃.

### Construction of pV190-***PDS*** and pV190-***TEN*** vectors

pV190 vector was kindly provided by Dr. Qinsheng Gu from Zheng Zhou Fruit Research Institute, Chinese Academy of Agricultural Sciences [12]. The pV190 vector was digested with BamHI and ligated with the target fragments with homology arms using homologous recombinase 2×Hieff Clone Enzyme Premix, and the ligated product was transferred into *E. coli* DH5α. Colony PCRs were conducted using the pV190 universal primer to screen for positive recombinant transformants. The positive clones were sent for DNA sequencing.

### ***Agrobacterium*** preparation and inoculation

The recombinant plasmid with the correct sequence was transformed into *Agrobacterium tumefaciens* GV3101. A single colony picked up from the agar plate was inoculated overnight in 1mL of YEP liquid medium containing 50 mg/L Kan and 25 mg/L Rif. A 100 µL aliquot of the above culture was added to 100 mL of new YEP liquid medium and incubated for 16–18 h up to OD_600_ 0.6–0.8. The cells were collected via centrifugation and re-suspended in a buffer containing 10 mM MgCl_2_, 10 mM MES, and 200 µM AS. For agroinfiltration, the suspension concentration of three bacteria containing pV190-*PDS*, pV190-*TEN* constructs, and negative control pV190 were adjusted to an OD_600_ of 0.8–1.0, with buffer as the blank control. The infection solution was maintained at room temperature for more than 2 h before inoculation.

Agroinfiltration was performed on seedlings with two true leaves. One or two small holes were gently made on the cotyledons and true leaves using a syringe needle. Each bacterial suspension was infiltrated into the leaves of plants through the small holes from the abaxial side of the leaf with a 1 mL needleless syringe. The plant trays were covered with clear polyethylene covers after infiltration to maintain a highly moist environment. The plants were cultured for 1 d under a dark condition at 24 °C and then under a photoperiod of 28 °C/24 °C and 16 h light/8 h dark and a relative humidity of 70% for further growth. The plants were not watered on the day of agroinfiltration but were watered from the following day.

### Total RNA extraction and expression analyses

To analyze the effect of VIGS on target gene expression, RNA was extracted from tissue samples that displayed a silencing phenotype. The controls were untreated tissues or tissues infected with a viral vector that did not carry the host gene segment insert. Each sampling time point for each treatment comprised three independent biological replicates.

50–100 mg leaf tissue was collect in a sterilized 2 mL centrifuge tube, and quickly frozen in liquid nitrogen. Total RNA was extracted and the first cDNA strand was synthesized according to the instructions of the Eastep^®^ Super Total RNA Extraction Kit (Promega, Beijing, China) and the Eastep® RT Master Mix Kit (Promega, BeiJing, China). Based on the cDNA sequence of *LaPDS* and *LaTEN* genes, specific fluorescent quantitative primers were designed, and *LaActin* was used as the internal reference gene. The real-time quantitative PCR (RT-qPCR) was carried out in CFX96 Real-Time System (Bio-Rad, CA, United States) with Eastep^®^ qPCR Master Mix (Promega, Beijing, China). The *Luffa* cDNA was diluted 5-fold as a template for RT-qPCR to detect the expression of targeted genes, with three replicates for each reaction. Relative expression levels were analyzed by 2^−△△CT^ of CT values [21]. Primer sequences for expression analysis are provided in the Additional file 1.

## Results

### Evaluation of TRV-VIGS efficiency in ridge gourd

**Fig. 1:**
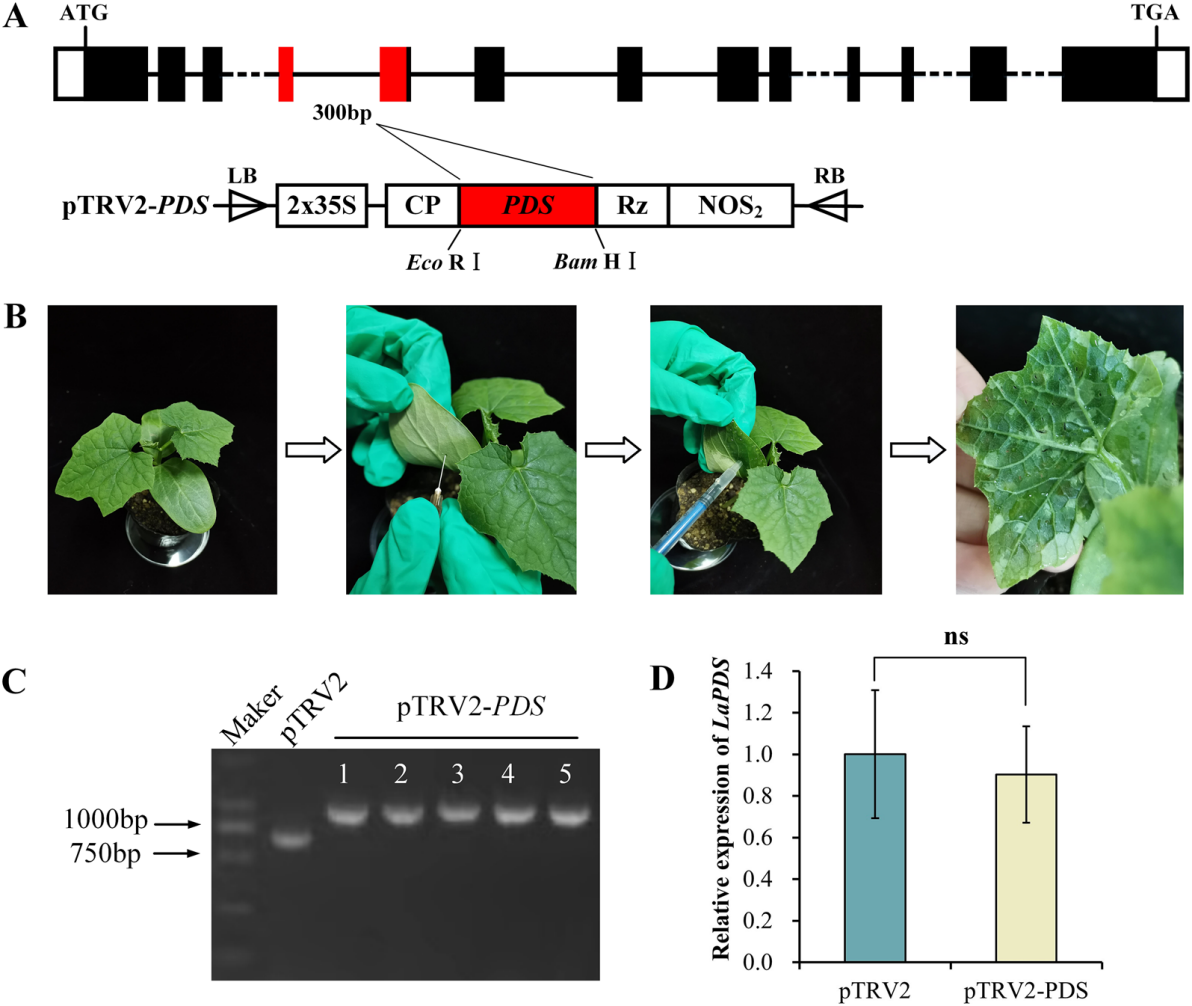
Evaluation of TRV-VIGS in ridge gourd using *PDS* gene. **A** *PDS* gene structure in ridge gourds and pTRV2-*PDS* vector map used in this study. White boxes represent 5′- and 3′-UTRs, black boxes represent exons, and the black line represents introns, red boxes represent target silent fragment. **B** Agroinfiltration of ridge gourd leaves. **C** Detection of pTRV2-*PDS* recombinant vector fragments after VIGS treatment (Lane 1 is Marker; Lane 2 is empty pTRV2 negative control; TRV RNA2 was detected in the upper newly emerged leaves tissue of ridge gourds infiltrated with pTRV2-*PDS Agrobacterium* suspension (Lane 3–7). **D** RT-qPCR analysis of *PDS* in ridge gourd leaves of inoculated with pTRV1 and pTRV2 or inoculated with pTRV1 and pTRV2-*PDS*. Error bar indicates SE. * Stands for significant difference (P < 0.05). n = 3 biological replicates

Tobacco rattle virus (TRV)-based virus-induced gene silencing (VIGS) is powerful functional genomics tool used in various plant species to for knocking down the expression of target genes in plants [22]. *PDS* is a rate-limiting enzyme involved in the carotenoid biosynthetic pathway [23]. Once *PDS* is successfully silenced, the young leaves of the plant will show albino symptoms. The phenotypic changes are obvious and easy to observe and distinguish; therefore, *PDS* can be used as a marker gene for VIGS in leaves [24]. To tested whether TRV clones could induce gene silencing in ridge gourd plants, we initially examined the ability of the TRV-VIGS vector to suppress the expression of the endogenous *PDS* in *L. acutangula*. A 300 bp fragment of the *PDS* gene was cloned into the TRV RNA2 vector (Fig. 1A). A mixture of *Agrobacterium* cultures containing pTRV2-*PDS* and pTRV1 was infiltrated into one true leaf and two cotyledons of the seedlings (Fig. 1B). However, the plants infected with pTRV2-*PDS* did not develop a photobleached phenotype on any leaf even 34 d post agroinfiltration. To further verify that *Luffa* seedlings were inoculated with the pTRV-*PDS* vector, inoculated plants were examined with the universal primers of the viral vector, the results showed that TRV RNA2 was detected in the tissues of the upper newly emergent leaves of ridge gourd treated with the pTRV2-*PDS Agrobacterium* suspension (Fig. 1C). We also used RT-qPCR to look for TRV infection in systemic leaves, and the results showed that there was no significant change in the degree to which *PDS* expression was silenced by the TRV vector in ridge gourd (Fig. 1D). Taken together, these findings indicate that, although the TRV-VIGS vector is capable of systemically infecting ridge gourd, it does not appear to trigger extensive gene silencing.

### Evaluation of CGMMV-VIGS efficiency in ***Luffa***

**Fig. 2:**
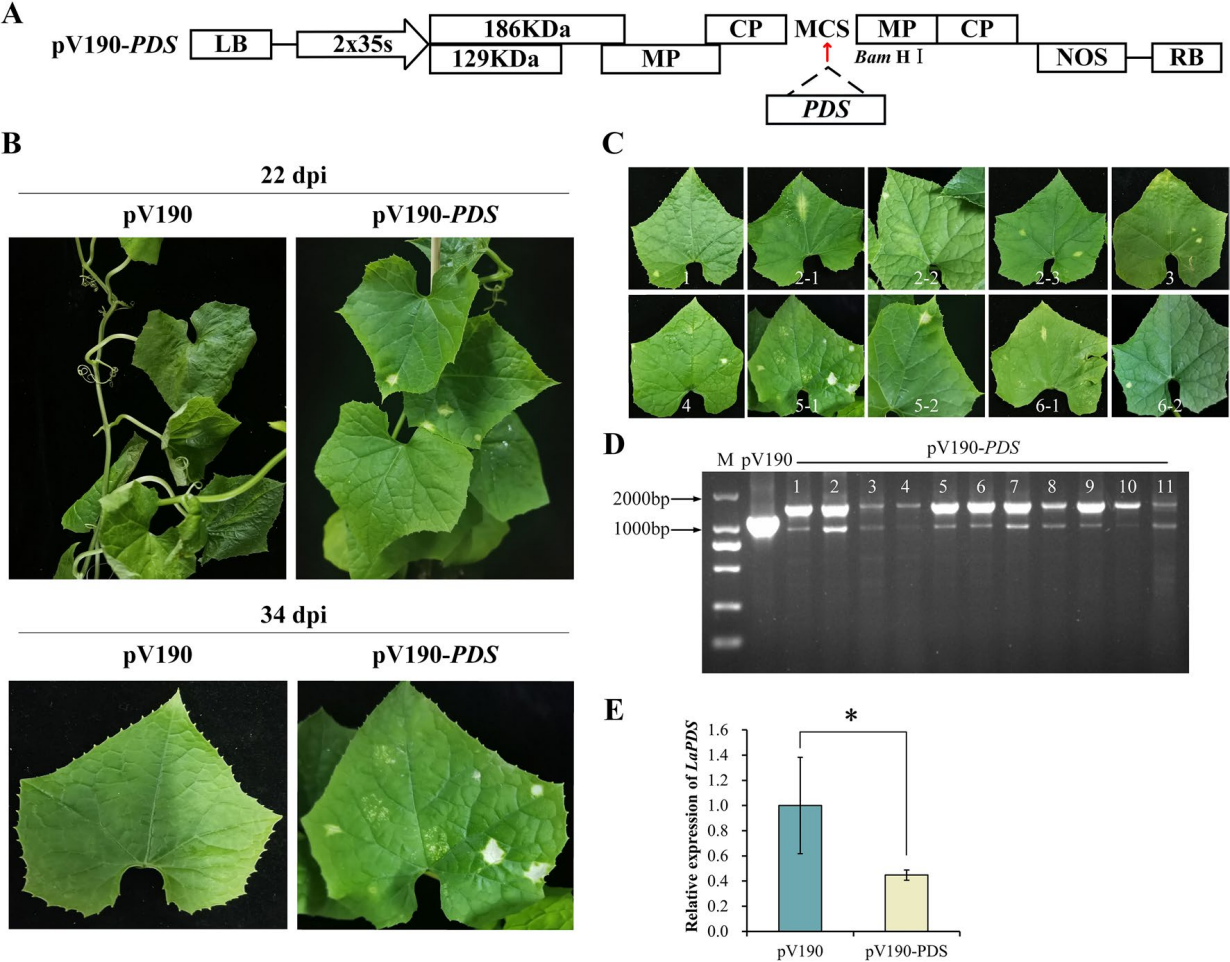
Silencing effect induced by CGMMV-VIGS vector in ridge gourd leaves. **A** The pV190-*PDS* vector map used in this study. **B** Photobleaching phenotype of ridge gourd leaves after inoculation with pV190-*PDS* for 22 d and 34 d. **C** Photobleaching phenotypes in different leaves of ridge gourd at 34 d. No. 2-1, 2-2, 2-3; 5-1, 5-2; 6-1, 6-2 represent different leaf positions on a single plant, whereas No. 1, 3 and 4 represent leaves from different plants. **D** Detection of pV190-*PDS* recombinant vector fragments after VIGS treatment [M: marker; Lane 1 is empty pV190 negative control; CGMMV RNA was detected in leaf tissues of ridge gourd that showed *PDS* silencing symptoms (lanes 2–12)]. **E** The relative expression of *PDS* after pV190-*PDS* recombinant vector was agroinfiltrated into ridge gourd leaves. Error bar indicates SE. * Stands for significant difference (P < 0.05). n = 3 biological replicates

Recently, a new VIGS vector derived from cucumber green mottle mosaic virus (CGMMV) was demonstrated to be a powerful and easy-to-use tool for characterizing the function of genes in several cucurbit crops [12]. Therefore, we tried this new vector on ridge gourd using the same method as above TRV-VIGS (Fig. 2A).

Leaves of plants inoculated with pV190-*PDS* began to exhibit photobleaching, whereas those of plants inoculated with pV190 did not undergo photobleaching at 22 dpi, indicating that the pV190-*PDS* vector effectively replicated and propagated in ridge gourd leaves and induced the silencing of *PDS* genes (Fig. 2B). Leaves exhibiting photobleaching were harvested from plants inoculated with pV190-*PDS*. Several leaves were taken from different positions on a single plant, whereas others derived from entirely different plants (Fig. 2C). To further verify that the *Luffa* seedlings were infected with the pV190-*PDS* vector, the albino phenotype plants were checked with the universal primers of the viral vector, and PCR results confirmed the presence of pV190-*PDS* transcripts in the leaves of the infiltrated plants (Fig. 2D), verifying the systematic infection of the virus. These results indicated that the pV190 vector effectively replicated and propagated itself in ridge gourd leaves and induced the silencing of marker genes.

To confirm the silencing of the *PDS* gene at the molecular level, leaves with the albino phenotype were collected from the plants with the photobleaching phenotype, and RT-qPCR results showed that the expression levels of the *PDS* gene decreased by 55% (Fig. 2E), indicating that gene silencing on the same plant was systematic.

### Silencing *LaTEN* in *Luffa* seedling

Tendrils are an important morphological feature of cucurbits, they have the ability to climb and attach, provide stable structural support for plants, and enable plants to obtain better living conditions [25]. *TEN*, which encodes a CYC/TB1-like transcription factor and plays a core regulatory role in plants, was identified to control tendrils [26]. We obtained the *LaTEN* gene from *Luffa* via homologous cloning. The photobleaching phenotype mainly appeared on the leaves. To further verify the utility of CGMMV-VIGS in other plant organs and tissues, we silenced the *LaTEN* gene for tendril development in *Luffa* seedlings.

**Fig. 3:**
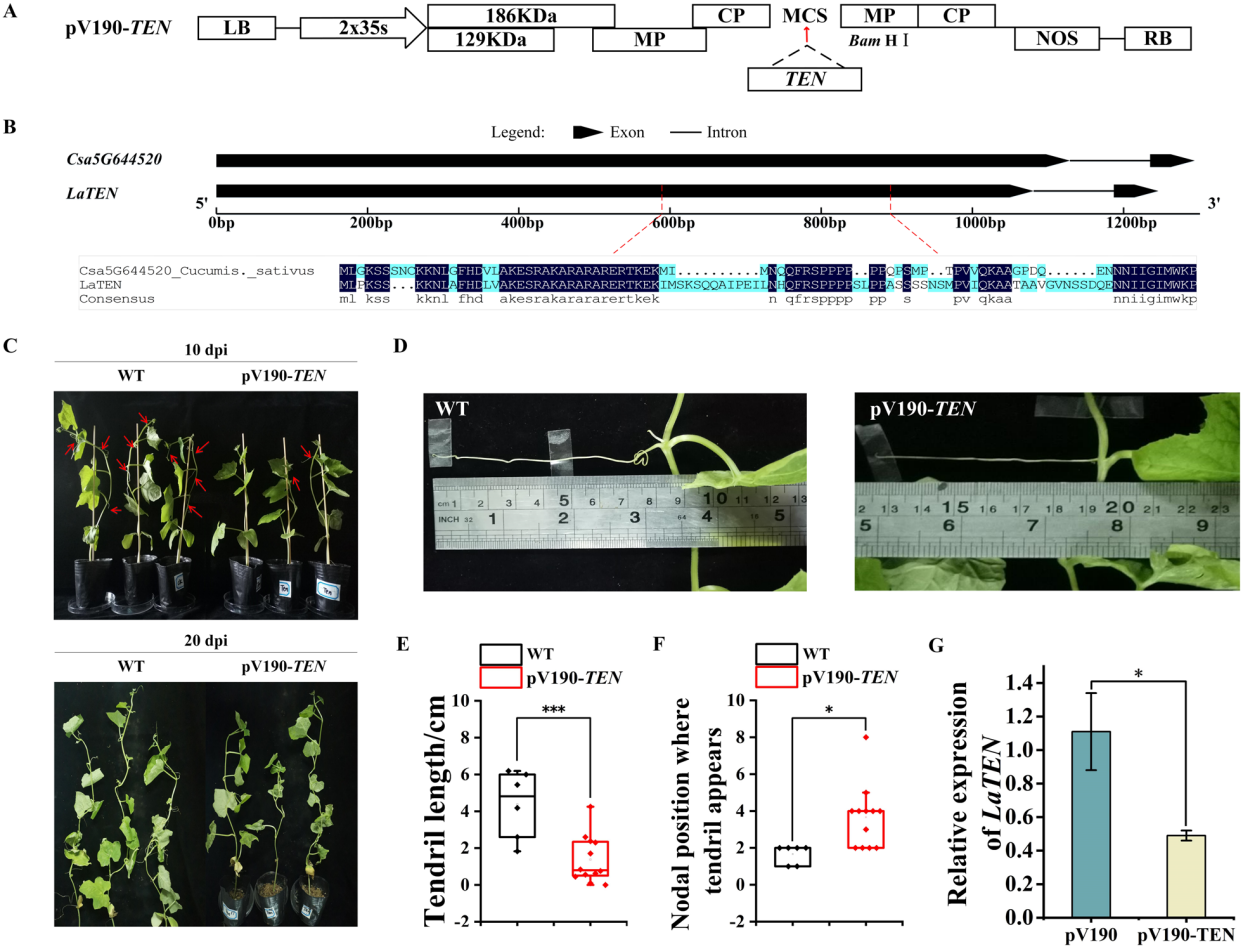
The utility of CGMMV-VIGS in ridge gourd using *TEN* gene. **A** The pV190-*TEN* vector map used in this study. **B** Structure diagram of cucumber and *Luffa TEN* gene; amino acid alignment of the *TEN* partial sequence of ridge gourd cloned for silencing; and the sequence of cucumber *TEN* gene. **C** Phenotype of plants with Wild type plants (WT) and infected pV190-*TEN* recombinant vectors at 10 and 20 dpi. **D** Length of tendrils at the fourth node of WT; length of tendrils at the third node of pV190-*TEN* recombinant vector-infected plants. **E**, **F** Length of tendrils and number of nodes of tendrils in plants with WT and infected pV190-*TEN* recombinant vectors at 20 dpi (due to the different nodes of each plant, the length of tendrils = the sum of the lengths of tendrils at each node/the number of nodes in the plant). **G** Relative expression levels of *TEN* genes after the pV190-*TEN* recombinant vector was agroinfiltrated into ridge gourd leaves. *** Stands for significant difference (P ＜0.001)

We performed phylogenetic analysis using the alignment of the closest homologues in the Cucurbit Genome Database (http://www.cucurbitgenomics.org/) and DNAMAN software, homology tree showed that from The *TEN* of the ridge gourd have the closest phylogenetic relationship to the homologues from the sponge gourd and pumpkin (Additional file 1: Figs. S1 , S2). The *LaTEN* gene was obtained in ridge gourd by blasting the cucumber *TEN* gene sequence with the local transcript data of ‘L422’ and designing primers that span a cDNA region of approximately 300 bp (Fig. 3B). The nodes where tendrils appeared and the length of tendrils at each node in the plants were scored and measured at 10 dpi and 20 dpi. The number of tendrils in the silenced plants was significantly lower than that in the wild type plants at 10 dpi (Fig. 3C). In silenced plants, the first and second nodes had no tendrils, the length of the tendrils in the third node was approximately 7 cm, and the tendrils in the fourth node were yet to grow. For untreated plants, the first, second, and third nodes all had tendrils, and the length of the tendrils in the fourth node was approximately 8.5 cm (Fig. 3D). Compared with the wild type plants, the *LaTEN*-silenced plants developed tendrils much later, with shorter tendril length and nodal positions where tendrils appear are higher (Fig. 3E, F). RT-qPCR showed that the relative expression of the *TEN* gene in the silenced plants was significantly lower than that of the injected empty plants (Fig. 3G), indicating that CGMMV-VIGS could induce effective gene silencing in the stem of the ridge gourd.

### Evaluation of CGMMV-VIGS efficiency in three cucurbits

**Fig. 4:**
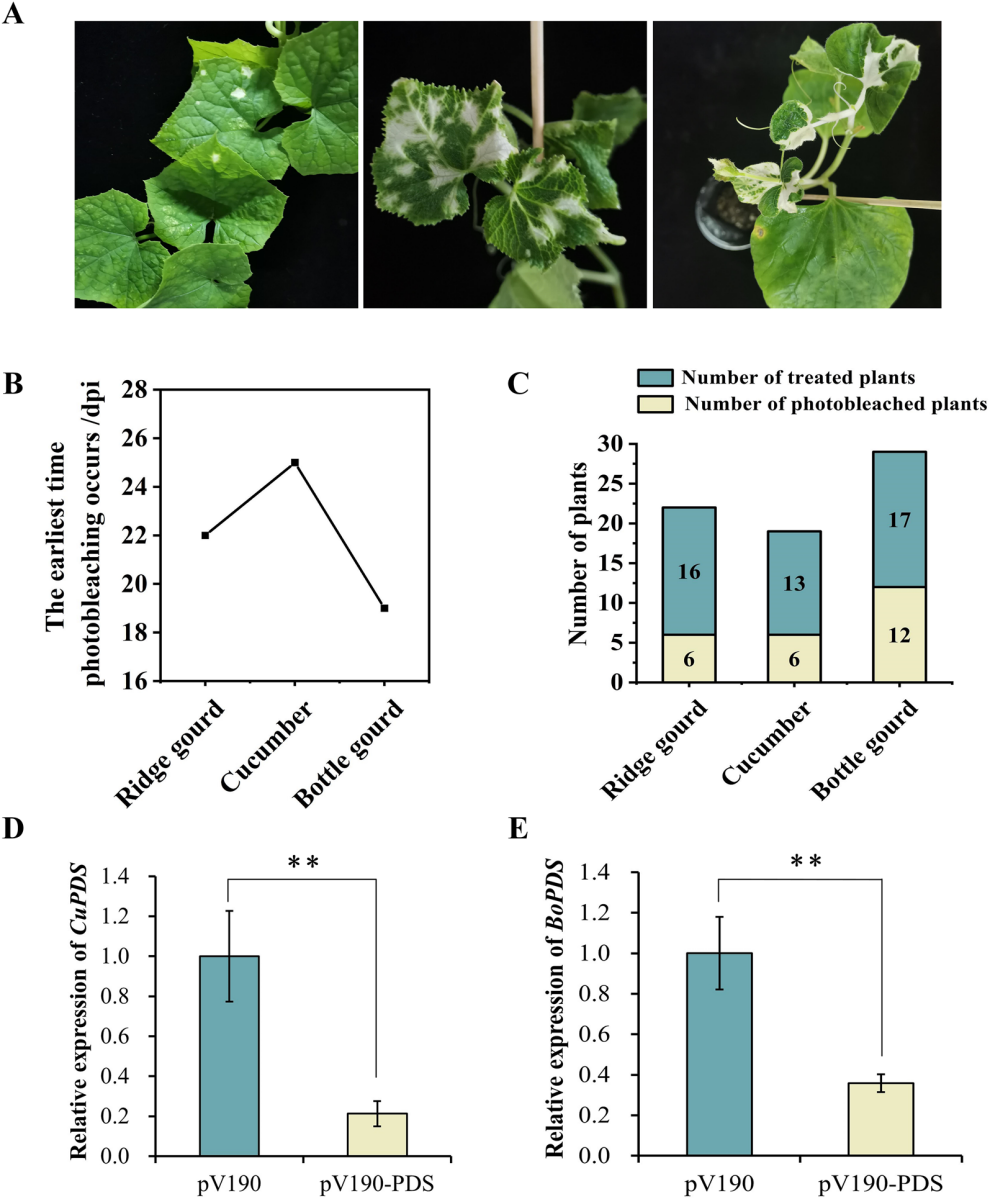
Evaluation of CGMMV-VIGS efficiency in three cucurbits. **A** Photobleaching phenotype of ridge gourd, cucumber, and bottle gourd after silencing the *PDS* genes with the CGMMV-VIGS vector. **B** The earliest time at which photobleaching occurred in three cucurbits. (**C**) Number of photobleached plants and treated plants in the three cucurbits. **D** and **E** The relative expression of the *PDS* gene after CGMMV-VIGS recombinant vector was agroinfiltrated into cucumber and bottle gourd leaves. Error bar indicates SE. ** indicates significant difference (P < 0.01)

Cucumber, ridge gourd and bottle gourd (*Lagenaria siceraria*) are three important vegetables in southern China. By targeting the same region of *PDS* genes in the three cucurbits, we compared the silencing efficiency of CGMMV-VIGS under the same environmental conditions (Fig. 4A). Alignment of amino acid sequences of *PDS* genes of cucumber, bottle gourd and ridge gourd are presented in Additional file 1: Fig. S3. The photobleached phenotype appeared on newly developed true leaves of the three crops three to four weeks after agroinfiltration (Fig. 4B). The bottle gourd was the most sensitive and exhibited the best silencing effect. Photobleaching occurred in 70.6% of the bottle gourd plants (12 of 17 plants). It occurred early at 19 dpi and became apparent at 24 dpi. In cucumber, photobleaching was visible at 25 dpi and became apparent at 31 dpi. Cucumber and bottle gourd leaves photobleached more than 50% of the total leaf area. *Luffa* was less sensitive to gene silencing, and photobleaching was visible at 22 dpi, slightly earlier than that in cucumber, but had a much lower bleaching coverage percentage than the other two crops (Figs. 2C, 4B, C; Table 1). The *PDS* gene expression level reduced to 45%, 21%, and 36% in ridge gourd, cucumber, and bottle gourd, respectively (Figs. 2E, 4D, E; Table 1).

**Table 1:** Comparison of *PDS* gene silencing in ridge gourd, cucumber, and bottle gourd

	Photobleaching phenotype		Intensity of photobleaching	PDS gene expression after VIGS treatment
Ridge gourd		34 dpi	+	45%
Cucumber		31 dpi	+++	21%
Bottle gourd		24 dpi	+++	36%

+ Intensity of photobleaching was visually estimated for each cultivar for the plant that showed the most intense silencing, and the percentage of photobleached leaf area to total leaf area was scored with plus signs: +, 10–20%; ++, 20–40%; +++, 40–60%

## Discussion

VIGS is a powerful tool for functional characterization of genes in plants; however, the application of VIGS is species-dependent. The silencing efficiency of VIGS largely depends on the interaction between the host plant and the virus and is closely related to the growth environment of the host and the environment in which the virus accumulates [27]. TRV is one of the most widely used vectors and has been successfully used in various plants [28]. Many studies have shown that the TRV vector is highly effective in all tested Solanaceae plants and less effective in some non-Solanaceae crops, such as rose [29]. This possibly due to differences in the virus host.

In the present study, no obvious silencing phenotype was observed after infecting ridge gourd with *Agrobacterium* carrying pTRV2-*PDS* (Fig. 1), but after changing to the vector pV190 modified from CGMMV, the silencing phenotype was induced (Fig. 2). The compatibility between the viral vector and the host plant also affects the silencing efficiency of VIGS. ALSV-VIGS could silence magnesium chelatase (*SU*) and phytoene desaturase (*PDS*) genes in four cucurbits, but it is not suitable for pumpkin [8].

We found that “the photobleaching area of ridge gourd was smaller than that of cucumber and bottle gourd, and the photobleaching phenotype in ridge gourd appeared later than that in bottle gourd,” which suggests that cucumber and bottle gourd are more sensitive to the CGMMV-VIGS system than is ridge gourd. Our results showed that photobleaching occurred not only on the leaves of bottle gourd but also on the petioles and stems. Previous research has shown that, in Zhejiang Province, CGMMV was initially isolated from bottle gourd and is typically propagated in bottle gourd (*Lagenaria siceraria*, Hangzhou chang gua) [30]. In this study, gourd was more sensitive to the pV190 vector, this may also be why gene silencing is more widespread in gourds, but not cucumber and ridge gourd. Furthermore, the chosen inoculation method also affects gene-silencing efficiency. We used a traditional syringe infiltration method to ensure introduction of the *Agrobacterium* suspension containing the recombinant silencing vector into the plant. This technique consisted of puncturing the backside of the cucumber leaf between the two veins with a needle to create a small hole, following which a needleless sterile 1 mL syringe containing the *Agrobacterium* suspension was pressed against the hole, allowing the suspension to slowly infiltrate into the leaf. As the suspension entered the intercellular space between the two leaf veins, the leaf tissue into which the suspension penetrated turned a deep green color.

CGMMV was confirmed to be associated with leaf green mosaic disease in *L. acutangula* [20]. Studies on agroinoculation of cDNA infectious clones in cucurbits with TRSV revealed that systemic infection of TRSV occurred in melon, oriental melon, and cucumber plants but not in watermelon and pumpkin [9]. Due to the economic importance of ridge gourd in south China, this study focused on one species of *Luffa*.

In addition, the photobleaching phenotype was not obvious when the seedlings were inoculated at the stage of two cotyledons probably because the plants were too small to resist the damage of the virus during inoculation, resulting in severe virus symptoms on the leaves, which affected the observation of the silencing phenotype (Additional file 1: Fig. S4). Therefore, we infiltrated two cotyledons and one true leaf with *Agrobacterium* at the stage of two true leaves. The photobleached phenotype appear on the 6th or 7th true leaves of *Luffa* plants infected with pV190-*PDS* 22 d after *Agrobacterium* injection (Fig. 2B) and the albino phenotype became very apparent 34 d after inoculation (Fig. 2B). RT-PCR showed that the plants infected with the pV190-*PDS* recombinant vector had two electrophoresis bands of the PCR product detected by the virus universal primer, and the size of the two bands differed by approximately 300 bp, which was the size of the target fragment. the leaves infected with recombinant virus vectors may contain both pV190-*PDS* transcripts in and pV190 empty vectors that partially lost the target fragment. The difference in band brightness may be due to the different silencing efficiency of each plant, resulting in different pV190-*PDS* transcripts in the inoculated leaves. If insert gene segments are too long, the virus may not spread or may lose the insert at a higher rate [27].

The *PDS* gene fragments of different lengths were inserted into the vector constructed based on PNRSV virus to infect *N. benthamiana*, and the stability of the inserted fragment was verified using RT-PCR. Photobleaching occurred when the length of the gene fragment was between 100 and 200 bp, whereas inserts larger than 200 bp of the target gene fragment were lost [31]. By inserting *PDS* gene fragments of different lengths into the CGMMV-modified viral vector, the inserted Cucurbitaceae *PDS* gene fragments with lengths 69, 150, 213, and 300 bp produced silencing phenotypes; however, RT-PCR showed that the 69 bp hairpin structure was not detected in the leaves, and leaves infected with a 300 bp *PDS* gene fragment contained varying degrees of fragment deletion [12].

Cucurbits are mostly cultivated by climbing the ground or artificially tying vines, and tendrils are redundant organs that consume nutrients. Therefore, tendrils need to be removed manually to reduce nutrient consumption and prevent nutrient demand competition between tendrils and fruits. Additionally, this assists in manually controlling the spatial distribution of plants and preventing the disorderly climbing and growth caused by tendrils. The removal of tendrils not only increases labor costs but also provides opportunities for the growth of pathogens from the wounds created after their removal. Therefore, tendril-free breeding is an important method that can be used to meet the needs of horticultural cultivation facilities. Cucurbitaceae tendrils have recently been identified as the source of stem (lateral branch) metamorphosis, and they are regulated by the key gene *TCP1* [32], which is a landmark research achievement in the regulation of plant tendrils. With the maturation of genetic transformation system and gene editing technology for cucurbit plants, precise editing and breeding without tendrils may be achieved [33]. Tendrils have little use for plant growth and development. Once tendrils encounter *Luffa* fruit, they climb and wrap around the fruit along the support due to chiral growth, resulting in a deformed shape of the *Luffa* fruit and affecting their yield and prices. We used the homologous cloning method to obtain the *LaTEN* gene in ridge gourd and preliminarily confirmed its function using the established VIGS system. The confirmed *LaTEN* could be a target gene for further editing to produce new varieties with fewer or no tendrils.

## Conclusion

We successfully established a CGMMV-based VIGS system on ridge gourd and used marker genes to identify the feasibility of the silencing system in *Luffa* leaves and stems. We also compared we evaluated the CGMMV-VIGS efficiency in three cucurbits, including Cucumber, ridge gourd and bottle gourd. The successful establishment of the *Luffa* VIGS system is of great significance in the study of *Luffa* functional genomics.

### Supplementary Information

**Additional file 1: Fig. S1.** Alignment of amino acid sequences of *TEN* genes of cucurbits. **Fig. S2. ***TEN* gene structure and homology tree in cucurbits. **Fig. S3.** Alignment of amino acid sequences of *PDS* genes of cucumber, bottle gourd and ridge gourd. **Fig. S4. **Agrobacterium infection of *Luffa* seedlings at cotyledon stage. **Table S1.** Primers used for constructing of VIGS vectors and RT-qPCR.

## Data Availability

The datasets used and/or analyzed during the current study are available from the corresponding authors on reasonable request.
