# Supplementary material for: Establishment of virus-induced gene silencing (VIGS) system in Luffa acutangula using Phytoene desaturase (PDS) and tendril synthesis related gene (TEN)
Source: Plant Methods. 2023 Aug 31;19:94. doi: 10.1186/s13007-023-01064-4 (PMC10470258; doi:10.1186/s13007-023-01064-4)
Supplement: Supplementary file 1 — Additional file 1: Fig. S1. Alignment of amino acid sequences of TEN genes of cucurbits. Fig. S2. TEN gene structure and homology tree in cucurbits. Fig. S3. Alignment of amino acid sequences of PDS genes of cucumber, bottle gourd and ridge gourd. Fig. S4. Agrobacterium infection of Luffa seedlings at cotyledon stage. Table S1. Primers used for constructing of VIGS vectors and RT-qPCR. [file 13007_2023_1064_MOESM1_ESM.docx]

| **Name** | **Sequence (5’-3’)** | **Application** |
| --- | --- | --- |
| TRV-LaPDS-F | CGGGATCCTTTGGGGCTTATCCCAA | VIGS vector |
| TRV-LaPDS-R | CGGAATTCTCTCATCCACTCTTGC | VIGS vector |
| pV190-LaPDS-F | CCCGTCAGGACTTTACTTAATGGATCCTTTGGGGCTTATCCCAA | VIGS vector |
| pV190-LaPDS-R | CGACCTAGACCTATAACTGGATCCTCTCATCCACTCTTGC | VIGS vector |
| qPCR-PDS-F | CGACCTAGACCTATAACTGGATCC | q-PCR |
| qPCR-PDS-R | CCAGTCTCATACCAGTCTCCATCAT | q-PCR |
| pV190-LaTEN-F | CCCGTCAGGACTTTACTTAATGGATCCTAATGTTGCCCAAGTCCTCA | VIGS vector |
| pV190-LaTEN-R | CGACCTAGACCTATAACTGGATCCTGGCTTCCACATGATCCCTA | VIGS vector |
| qPCR-TEN-F | AAGCCAGCAAAACCCTAGA | q-PCR |
| qPCR-TEN-R | TAACCTCACATTCAGAATCCAA | q-PCR |
| LaActin-F | GTACAACTGGTATCGTGCTG | q-PCR |
| LaActin-R | AGGTCCAAACGGAGAATT | q-PCR |
| PCR-pTRV2-F | TGAGGGAAAAGTAGAGAACG | VIGS vector |
| PCR-pTRV2-R | CCTATGGTAAGACAATGAGT | VIGS vector |
| PCR-pV190-F | TCCTTTGACTTTAGAGGTCG | VIGS vector |
| PCR-pV190-R | TGGGCCCCTACCCGGGGAA | VIGS vector |

Table S1. Primers used for constructing of VIGS vectors and RT-qPCR.

Figure S1. Alignment of amino acid sequences of *TEN* genes of cucurbits


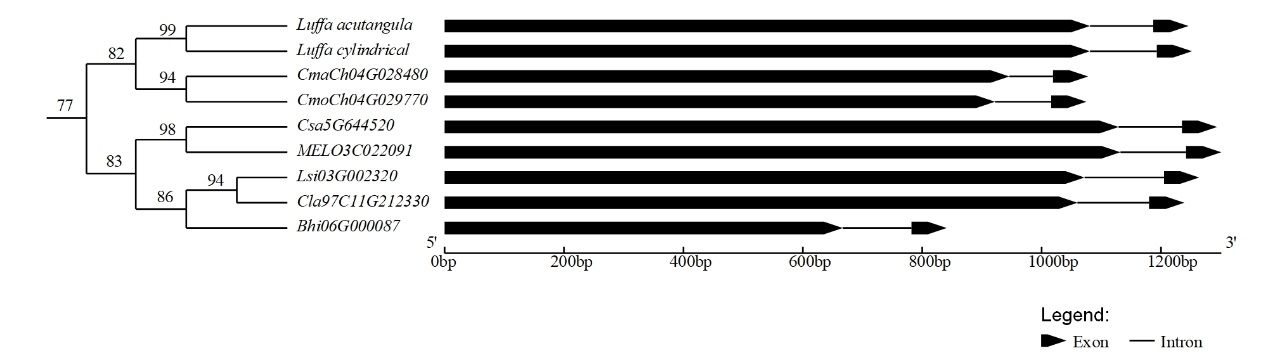
Figure S2. *TEN* gene structure and Homology Tree in Cucurbits

Figure S3. Alignment of amino acid sequences of *PDS* genes of cucumber, bottle gourd and ridge gourd


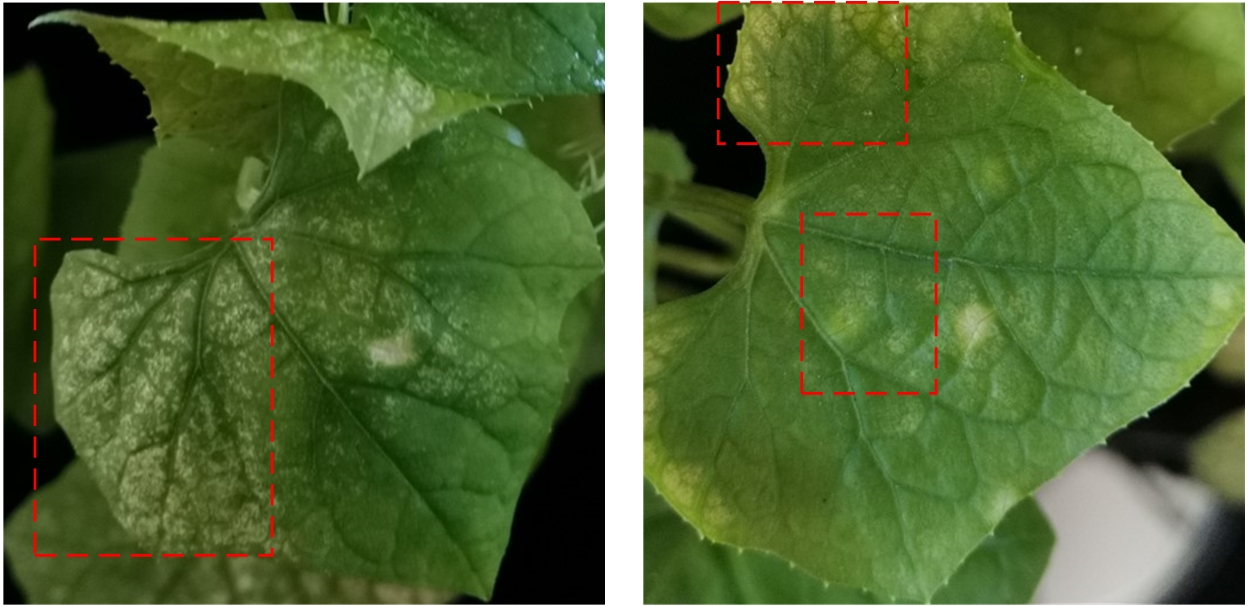


Figure S4. Agrobacterium Infection of Luffa Seedlings at Cotyledon Stage. Cotyledon injection 25dpi. The symptoms of the virus are more obvious, which affects the observation of the photobleaching phenotype.
